# Unintentional Recovery of Parasitic Diversity Following Restoration of Red Deer (*Cervus elaphus*) in North-Western Italy

**DOI:** 10.3390/ani12111433

**Published:** 2022-06-02

**Authors:** Barbara Moroni, Mattia Begovoeva, Luca Rossi, Samer Angelone, Serena Robetto, Luca Visconti, Anna Regis, Roberto Viganò, Nicole Preacco, Simona Zoppi, Luisa Rambozzi, Pier Giuseppe Meneguz

**Affiliations:** 1 Department of Veterinary Sciences, University of Turin, Largo Braccini 2, 10095 Grugliasco, TO, Italy; luca.rossi@unito.it (L.R.); luisa.rambozzi@unito.it (L.R.); piergiuseppe.meneguz@unito.it (P.G.M.); 2 Istituto Zooprofilattico Sperimentale di Piemonte, Liguria e Valle d’Aosta, Via Bologna 148, 10154 Torino, TO, Italy; simona.zoppi@izsto.it; 3 European Commission for the Control of Foot-and-Mouth Disease, Food and Agriculture Organization of the United Nations, Viale delle Terme di Caracalla, 00153 Rome, RM, Italy; mattia.begovoeva@fao.org; 4 Department of Evolutionary Biology and Environmental Studies, University of Zurich, Winterthurerstrasse 190, 8057 Zurich, Switzerland; samer.angelone@ieu.uzh.ch; 5 Istituto Zooprofilattico Sperimentale Piemonte, Liguria e Valle d’Aosta, Centro di Referenza Nazionale Malattie Animali Selvatici (CeRMAS), S.C. Valle d’Aosta- S.S. Patologie della Fauna Selvatica, Rue de l’Amerique 7G, 11020 Quart, AO, Italy; serena.robetto@izsto.it; 6 Independent Researcher, 21036 Gemonio, VA, Italy; lucavisconti.vet@virgilio.it; 7 Independent Researcher, 10050 Bruzolo, TO, Italy; an.regis93@gmail.com; 8 Independent Researcher, 21052 Busto Arsizio, VA, Italy; r.vigano@alpvet.it; 9 Independent Researcher, 13878 Candelo, BI, Italy; nicole@nickland.org

**Keywords:** red deer, wildlife, translocations, nasal bot, warble fly, nodular onchocercosis, parasitic biodiversity

## Abstract

**Simple Summary:**

In the early sixties, free ranging red deer (*Cervus elaphus*, L.) were absent in Piedmont. Human-driven translocations and spontaneous migration of red deer from Switzerland and France resulted in the successful redistribution of this wild ungulate. In parallel, host-specific parasites harbored by red deer populations disappeared in the same area until the restoration of red deer in north-western Italy. The parasitic community has been enriched with at least two species-specific taxa, *Onchocerca jakutensis* and *Pharyngomyia picta*, suggesting that the recovery of parasitic biodiversity could be included amongst future conservation goals of this intensively managed game.

**Abstract:**

Red deer (*Cervus elaphus*) populations in north-western Italy have been remodeled in recent decades. Multiple translocations and the spontaneous migration from Switzerland and France resulted in the successful redistribution of the red deer after human-driven extirpation during the 18th century. The scarcely diverse parasitic community harbored by these cervids has been enriched with two species-specific taxa, *Onchocerca jakutensis* and *Phayigomyia picta*, suggesting that the recovery of parasitic biodiversity could be included amongst future conservation goals of this intensively managed game. Nodular onchocercosis was reported in three red deer populations since 2011, while nasal bots were reported since 2018. *Hypoderma* spp. larvae were identified for the first time in 1989, then a second record was made in 2014 in the province of Biella, where a yearling male in poor condition infested with *Hypoderma diana* was observed. In the perspective that the restoration of species-specific parasite communities of native mammals in Europe is increasingly perceived as a conservation target, with similar dignity as the conservation of their hosts, baseline data presented in this communication may give new insights for future parasite conservation efforts.

## 1. Introduction

Translocations have been and are still extensively implemented as a tool for the management and restoration of biodiversity [1,2]. Such interventions have often made a difference, by permitting and/or accelerating the recovery of native species on a geographical scale, making them extirpated or critically rarified. The red deer (*Cervus elaphus*) is amongst the large mammalian species which greatly benefitted from translocation policies in Europe and Italy [3,4].

In Conservation Medicine, a lively debate exists on the priority to assign to parasitic biodiversity as a conservation target aside their hosts [5]. Awareness has undoubtedly grown over recent decades on the role of wildlife–parasite relationships as fundamental drivers of ecological structure and function, and the eco-systemic services that parasites are able to provide in the long term [6]. Nevertheless, despite the cultural evolution in progress, evidence from the field shows that parasitic biodiversity is still far from receiving the positive attention it deserves from the conservation community, resulting in the unintentional or sometimes deliberate loss of wildlife parasites at several steps of conservation programs [7]. This short communication aims to illustrate the unintentional recovery of parasitic biodiversity in a large mammal model. In north-western (NW) Italy, multiple translocations over 50 years and the spontaneous migration from neighboring countries (Switzerland and France, where multiple translocations have been also carried out) resulted in the successful redistribution of the red deer (hereafter RD) after human-driven extirpation during the 18th century [3]. In parallel, the scarcely diverse parasitic community initially harbored by these deer was enriched with (at least) two species-specific taxa, *Onchocerca jakutensis* and *Pharygomyia picta*, suggesting that the recovery of parasitic biodiversity could be included amongst future conservation goals of this intensively managed game.

## 2. Materials and Methods

**Figure 1:**
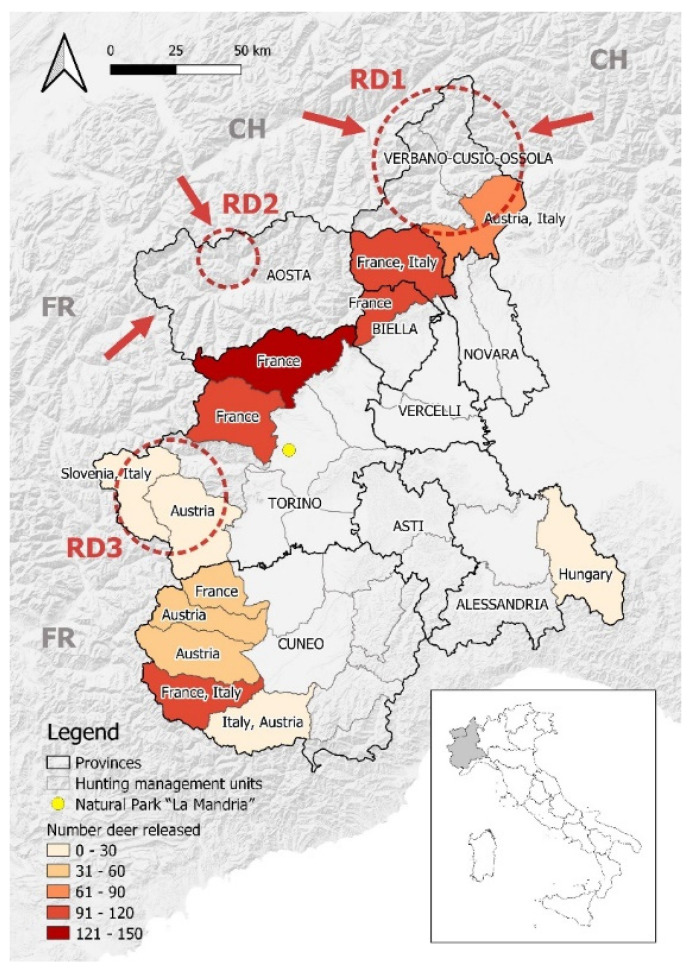
Map of NW Italy showing the hunting management units where RD were translocated between 1962 and 2005. The red circles highlight the areas under passive surveillance for macroscopic external parasites (RD1 = Ossola Valleys; RD2 = Gran S. Bernardo and Valpelline Valleys; RD3 = Susa and Chisone-Germanasca Valleys). Arrows indicate the zones that were initially recolonized by RD migrated from Switzerland (CH) and France (FR). The yellow dot corresponds to the Natural Park of La Mandria. Names of the country in the colored areas represent the origin country of the RD population. Borders of provinces adapted from: Regione Piemonte—A1613B—Sistema informativo territoriale e ambientale (https://www.geoportale.piemonte.it/geonetwork/srv/eng/catalog.search#/metadata/r_piemon:94c85f56-4755-470a-8587-f4644b19ccbd, accessed on 10 March 2022). Borders of hunting management units adapted from: Regione Piemonte—A1709C—Infrastrutture, territorio rurale, calamità naturali in agricoltura, caccia e pesca (https://www.geoportale.piemonte.it/geonetwork/srv/eng/catalog.search#/metadata/r_piemon:d4e3df50-bc23-4be5-8825-b9b2c4b218fc, accessed on 10 March 2022). Hill shades adapted from: OpenMapTiles.org © MapTiler © OpenStreetMap contributors.

Since 1978, the Parasitology research unit at the Department of Veterinary Sciences of Turin, Italy, was involved in the passive surveillance of RD mortality causes in Northwestern Italy (Piedmont and Aosta Valley regions) and the inspection of RD carcasses in three areas (hereafter named RD1, RD2, RD3), where hunting had become legal practice from the mid-eighties onward (Figure 1). Within this frame, particular attention was devoted to recording the presence of macroscopically detectable metazoan parasites affecting the skin and the upper respiratory tract, including warbles (*Hypoderma* spp.), nasal bots (*Oestridae*), and the nematode agents of subcutaneous onchocercosis (*Onchocerca* spp.). Accordingly, data and images presented in this short communication are to be understood as taken from the archives of the forementioned Parasitology Research Unit. The index records at the local level prompted further investigation aimed to finetune the identification at the species level and deepen the knowledge on the distribution of the new parasites, by interview of game professionals and consultation of the culling cards in their databases. Local records were finally cross-matched with the movements of the affected and neighboring populations of RD and other sympatric cervid hosts (roe deer *Capreolus capreolus*, and fallow deer *Cervus dama*) to identify possible sources of the investigated parasites. Data on RD populations in this study (Table 1 and Figure 1) were obtained from unpublished reports and the RD management data archive of one of the authors (PGM). Complementary surveys included the key-guided morphological identification of new parasites [8,9,10], the molecular identification of warbles and *Onchocerca* specimens by means of mitochondrial markers [9,11], a serosurvey for anti-*Hypoderma* antibodies [12], and the drafting of thematic maps of the distribution of infected RD. Maps were developed using QGIS software 3.2.0 “Bonn”.

## 3. Results

The origin, translocation year, and number of translocated RD are shown in Figure 1 and Table 1.

**Figure 2:**
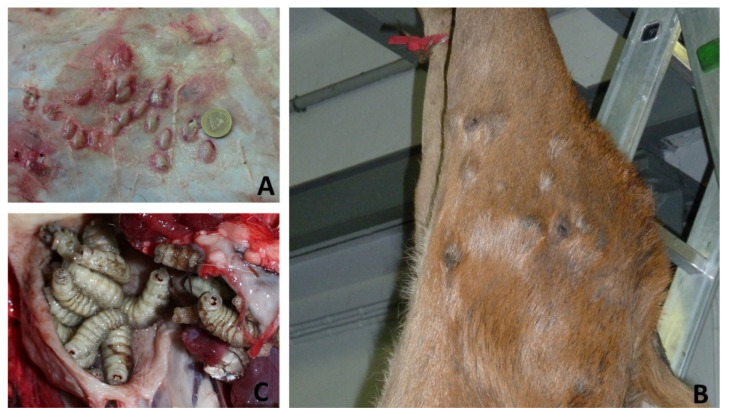
Macroscopic view on *Hypoderma diana* (**A**), *Onchocerca jakutensis* nodules (**B**), *Pharyngomya picta* (**C**) on deer from different areas of Piedmont (see Figure 3).

*Hypoderma* spp. larvae were identified for the first time in 1989, in the hinds of two RD (of a group of ten) that did not survive the transport stress from Hungary (Figure 2).

Ever since, no warbles were found in 82 descendant RD (culled since 2010) and the abundant sympatric fallow deer (on average, 171 individuals culled per hunting season between 1997 and 2021). A second record concerned a yearling male in poor condition, culled in the Biella province in 2014. Several tens of third stage larvae were observed and a sample of them was morphologically and molecularly attributed to *Hypoderma diana* (Figure 2A). No warbles were recorded in 170 RD inspected in the province of Biella between 2013 and 2019. However, 7 out of 35 (20%) serum samples obtained from these RD in 2013-2015 tested positive for *Hypoderma* spp. antibodies. Interestingly, in those years (starting from 2011 with the first positive roe deer found in Ivrea, province of Turin), warbles by *H. diana* were frequent finding in sympatric roe deer.

**Figure 3:**
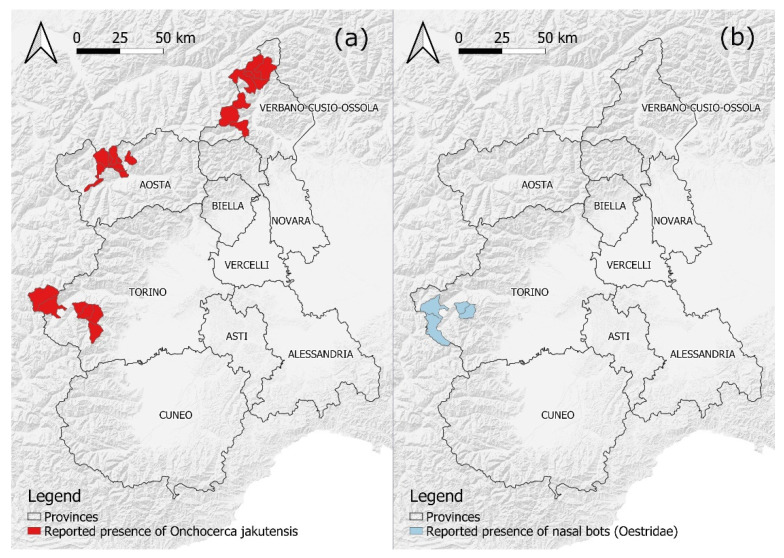
Location (by municipality) of the cases of nodular onchocercosis reported in the surveyed RD populations since the first detection in 2011 (**a**), and cases of nasal bot infestation in the surveyed RD populations since the first detection in 2018 (**b**). Borders of provinces adapted from: Regione Piemonte—A1613B—Sistema informativo territoriale e ambientale (https://www.geoportale.piemonte.it/geonetwork/srv/eng/catalog.search#/metadata/r_piemon:94c85f56-4755-470a-8587-f4644b19ccbd, accessed on 10 March 2022). Hill shades adapted from: OpenMapTiles.org © MapTiler © OpenStreetMap contributors.

Nodular onchocercosis was reported in three RD populations, since 2011 in RD1, 2012 in RD 3, and 2013 in RD2, respectively (Figure 2B). Shortly before, in 2010, one of the authors (LV) reported *Onchocerca* nodules in RD culled in the nearby province of Varese, located in the Lombardy region at the border with Canton Ticino, Switzerland. Nematodes of both sexes, isolated by dissecting the nodules on the thighs of affected RD originating from all areas, were morphologically identified as *Onchocerca jakutensis* (syn. *O. tubingensis*). The diagnosis was molecularly confirmed in six specimens collected on culled individuals. After the local index case, other cases were recorded in the subsequent years (Figure 3) The apparent yearly prevalence in hunted RD ranged from 0.5 to 25%, with the highest values observed in RD1, where passive surveillance data were complemented with information provided by professional slaughterers responsible for the skinning of RD in the frame of a study on a local venison supply chain [13]. Cases were signaled in individuals of all ages, with the remarkable exception of calves.

Nasal bots were reported since 2018 in a limited number of RD culled in RD3 (Figure 3b). Second- and third-stage larvae collected from four of ten infected individuals were morphologically attributed to *Pharyngomyia picta*. The kidney-shaped appearance of posterior spiracles and the clearly separated fleshy cones of the pseudocephalon were particularly considered for the differential diagnosis with *Cephenemyia* spp., according to de La Fuente [14] and Colwell [15]. In the nineties, a high prevalence of *P. picta* and *C. auribarbis* infection was recorded in a closed population of RD in the Natural Park of La Mandria, on the outskirts of Turin city (196 infected individuals out of 401 sampled; 49%), whereas nasal bots were not found at those times in the heads of 27 RD from RD3 [16].

## 4. Discussion

The history of RD restoration in NW Italy, and the long-term surveillance of selected parasitic infections (namely those with an obvious macroscopic presentation) in a sample of the RD populations established in the study area, offered us a unique perspective on co-restoration of RD-specific metazoans following the translocation and/or spontaneous migration of the host from neighboring zones via faunal corridors. The unintentional and (less frequently) intentional restoration of parasitic biodiversity in the frame of conservation projects has been reported in other mammalian models throughout the last few decades [17,18,19,20], while the recovery of generalist parasites in spontaneous wildlife reintroduction is more common [21,22].

In the present study, none of the investigated RD-specific parasites were present in RD3 at 25 to 30 years after reintroduction (Table 1). This may be attributed to one or a combination of the following events [23]: (i) the few founders translocated from Slovenia did not harbor the parasites; and/or (ii) the parasites became extinct in the reintroduction site, eventually due to failure by adult Oestrid flies or *O. jakutensis* vectors (simuliids and ceratopogonids), in encountering suitable hosts at a low host population size. Both *P. picta* and *O. jakutensis* are known to be endemically present in Slovenia [24], but no parasite check was conducted at release. However, only eight individuals safely survived to transport and release stress. To the best of our knowledge, limited information is available on the persistence and spread of specific Oestrid flies or Onchocercinae following the translocation of infected founder deer. Interestingly, however, nasal bots did not become established in RD in New Zealand, despite the introduction (between 1861 and 1926) of more than 250 founder individuals mostly originating from Scotland, UK, where *C. auribarbis* was traditionally endemic [25]. Based on the above, it is reasonable to assume that the mere translocation of RD founders infested by Oestrid larvae does not guarantee that these parasites will become established in the host population that will stem, especially when few individuals are liberated.

*Onchocerca jakutensis* (syn. *O. tubingensis*) is one of the four *Onchocerca* representatives that may develop in the RD, and one of the two (the other is *O. flexuosa*) that result in macroscopic subcutaneous nodules, typically developing on the thighs and gluteus region of affected individuals [26]. This filariid nematode has been endemically reported in Eastern and Central Europe, including Germany, Austria, the former Yugoslavia, Czech Republic, Slovak Republic, Poland, and Romania [11]. In Italy, there is a single report of *O. jakutensis* in RD in Tuscany, Central Italy [11]. It is noteworthy that these deer originated from only seven founders translocated in the early sixties from the Tarvisio State Forest, a conservation hotspot previously re-colonized by migrated deer from neighboring Austria and Slovenia. In 2013, *O. jakutensis* was reported in Switzerland for the first time [24]. Just like in Italy, the RD was extirpated from the country during the 19th century, then returned following spontaneous migration from Austria and a few translocations (e.g., from Carinthia, Austria, to Canton Valais). The recent spread of *O. jakutensis* in Switzerland can likely explain the records in RD2 and possibly RD3, since both populations are historically connected with the ones in southern Switzerland and actually derived from them. As an alternative hypothesis, *O. jakutensis* was introduced in RD3 in 2002, with 80 RD translocated from a farm in Carinthia, Austria. In both RD2 and RD3, the parasite detection was unlikely until relatively recently, when RD hunting became legal practice and trained staff were enrolled to inspect all carcasses (in 2012 in RD2 and 2005 in RD3). Regarding RD1, where RD is hunted and professionally inspected since 1986, evidence shows that *O. jakutensis* was not introduced by the few founder RD translocated in the early sixties. However, Figure 1 and Table 1 show that a restocking intervention was carried out in 2002 at the south-easternmost corner of RD1 involving 25 individuals originating from Carinthia; moreover, 104 RD originating from Chambord, France, were reintroduced north of RD1 in 2004–2005. Given the distribution of *O. jakutensis* (endemic in Austria and apparently absent in France) and the location of the first records of nodular onchocercosis observed in RD1, it is reasonable to assume that this filariid nematode was introduced for the former restocking intervention. The time interval from the reintroduction of these RD in 2002 and the first report of *O. jakutensis* in RD1 in 2013 is consistent with the dispersal pattern of a marked sample of translocated individuals [27] and the limited flight range of *Onchocerca* vectors [28,29].

Together with *C. auribarbis*, *P. picta* is one of the two host-specific nasal bots infecting RD [15]. A third species, *Cephenemyia stimulator*, is sporadically recorded in RD living in sympatry with infected roe deer (*Capreolus capreolus* L.) [30]. Due to spatial proximity and temporal coherence, the origin of *P. picta* in RD3 (Figure 1) may be reasonably traced back to the relatively recent translocation of deer from Carinthia, Austria, for restocking purposes (Table 1). *P. picta* has been retrieved in approximately half the RD sampled in Vorarlberg and Tyrol, Western Austria, at the borders with Carinthia, whereas the co-infecting *C. auribarbis* showed a lower prevalence of 8% [31]. This may justify why the latter nasal bot did not become established in RD3. Based on the literature, RD-specific nasal bots are absent in France and southern Switzerland, thus justifying the absence of records from other surveilled locations in northwestern Italy. Interestingly, the endemic occurrence of *C. auribarbis* ad *P. picta* in the closed deer population on the outskirts of Turin city did not result in the infection of other free-ranging RD within the study area, despite the remarkable flight range (in the order of several tens of kms) that characterizes some Oestrids [15]. Regrettably, no published information seems to be available on the dispersal of adult *C. auribarbis* and *P. picta*. Finally, neither *O. jakutensis* nor nasal bots were recorded amongst descendants of the numerous RD (>500) translocated from Chambord, France, suggesting that founders with that origin did not contribute to the restoration of RD-specific parasitic diversity in the study area. Accordingly, future surveillance for RD-specific nasal bots in NW Italy will be preferably enforced in zones whose RD derived (or partially derived) from Austria (Table 1).

Results of this study show the merits and also the limits of passive surveillance for the tracking of “new entries” in the parasite communities in wildlife. In particular, we experienced that, even in a professionally and regularly monitored game species, several years may elapse from the introduction of putatively infected hosts to the first signaling of easy-to-diagnose macro-parasites, such as *O. jakutensis* and *P. picta*. The delay may be attributed to a mix of factors, including randomness, their low initial prevalence, the time taken by new parasites to become established in “core” zones where the majority of deer seasonally congregate (e.g., during fall/early winter), the abundance of vectors in RD habitat, the vectors’ flight performance, the awareness of hunters, etc.

Typical bottlenecks of parasitic biodiversity are those reintroductions in which founders, whether raised in captivity or obtained from wild stocks, are translocated in limited number and eventually subjected to treatments with broad-spectrum antiparasitic agents to improve individual fitness and thus favor intervention success [32]. Under these circumstances, species-specific parasites may either not be translocated with the host or become locally extinct due to post-release transmission constraints, eventually related to the low abundance of translocated founders and their unpredictable social/spatial behavior [23].

In the perspective that the restoration of species-specific parasite communities of native mammals in Europe is increasingly perceived as a conservation target, with similar dignity as the conservation of their hosts [7,33], cues may be drawn from this study to improve future parasite conservation efforts as follows: (i) the species-specific parasite community of any mammal species to reintroduce or reinforce should be known in advance; (ii) all other guarantees being equal (e.g., lineage, genetic variability, freedom from pathogens of major significance for human and livestock health, etc.), whereby founders should preferably originate from populations/zones enjoying a high diversity of specific parasites; (iii) should any species-specific parasitic group be missing in the restored host population, restocking can be carried out with individuals originating from populations endemically harboring the missing parasites; and iv) to check if the restoration of parasitic biodiversity has met the expectations, passive surveillance by trained staff should be complemented with active surveillance, including a realistic sampling design and the application of diagnostic techniques suitable for the target parasites and host conservation status.

## Figures and Tables

**Table 1:** Origin and number of red deer translocated to Piedmont between 1962 and 2005.

Reintroduction Area (Province)	Year	Origin	Number of RD
Susa Valley (Torino)	1962–1964	Kocevje (Slovenia)	12
		Zoo park, Cuneo (Italy)	3
Spinti Valley (Alessandria)	1989	Deer farm (Hungary)	10
Pesio Valley (Cuneo)	1990	Wild park, Paneveggio (Italy)	3
	1991–1993	Tarvisio State Forest (Italy)	7
	1995	Wild park, Tyrol (Austria)	3
Stura Valley (Cuneo)	1989	Zoo park, Novara (Italy)	4
	1990–1998	Chambord (France)	116
Sesia Valley (Vercelli)	1995	Susa Valley (Italy)	4
Sesia/SesseraValleys (Vercelli/Biella)	1997	Chambord (France)	108
Po Valley (Cuneo)	1998–2000	Chambord (France)	59
Strona/ Cannobina Valleys (Verbania)	2001	Zoo park, Poppi (Italy)	4
	2002	Carinthia (Austria)	80
Chisone Valley (Torino)	2002	Carinthia (Austria)	25
Maira Valley (Cuneo)	2002	Carinthia (Austria)	41
Varaita Valley (Cuneo)	2002	Carinthia (Austria)	40
Lanzo Valley (Torino)	2004–2005	Chambord (France)	104
Orco/SoanaValleys (Torino)	2002–2005	Chambord (France)	149

## Data Availability

All the data presented in this study are included within the article. Further inquiries can be directed to the corresponding author.

## References

[1] Seddon P.J., Strauss W.M., Innes J. (2012). Animal translocations: What are they and why do we do them. Reintroduction Biol. Integr. Sci. Manag..

[2] Furlan E.M., Gruber B., Attard C.R.M., Wager R.N.E., Kerezsy A., Faulks L.K., Beheregaray L.B., Unmack P.J. (2020). Assessing the benefits and risks of translocations in depauperate species: A theoretical framework with an empirical validation. J. Appl. Ecol..

[3] Mattioli S., Meneguz P.G., Brugnoli A., Nicoloso S. (2001). Red Deer in Italy: Recent Changes in Range and Numbers. Hystrix.

[4] Putman R., Apollonio M., Andersen R. (2011). Ungulate Management in Europe: Problems and Practices.

[5] Gómez A., Nichols E. (2013). Neglected wild life: Parasitic biodiversity as a conservation target. Int. J. Parasitol. Parasites Wildl..

[6] Hudson P.J., Dobson A.P., Lafferty K.D. (2006). Is a healthy ecosystem one that is rich in parasites?. Trends Ecol. Evol..

[7] Carlson C.J., Hopkins S., Bell K.C., Doña J., Godfrey S.S., Kwak M.L., Lafferty K.D., Moir M.L., Speer K.A., Strona G. (2020). A global parasite conservation plan. Biol. Conserv..

[8] Draber-Mońko A. Morphologie einiger fliegenlarven der familie oestridae (Diptera). Proceedings of the Annales Zoologici.

[9] Otranto D., Traversa D., Guida B., Tarsitano E., Fiorente P., Stevens J.R. (2003). Molecular characterization of the mitochondrial cytochrome oxidase I gene of Oestridae species causing obligate myiasis. Med. Vet. Entomol..

[10] Demiaszkiewicz A.W. (1993). Redescription of *Onchocerca jakutensis* (Gubanov, 1964) (Nematoda, Filarioidea). Acta Parasitol..

[11] Morandi F., Krueger A., Panarese S., Sarli G., Verin R., Nicoloso S., Benazzi C., Galuppi R. (2011). First description of nodular onchocercosis (*Onchocerca jakutensis*) in free-ranging Italian red deer (*Cervus elaphus*). J. Wildl. Dis..

[12] Panadero R., Martínez-Carrasco C., León-Vizcaíno L., López C., Díez-Baños P., Morrondo M.P., Alonso F. (2010). Use of a crude extract or purified antigen from first-instar cattle grubs, Hypoderma lineatum, for the detection of anti-Hypoderma antibodies in free-ranging cervids from southern Spain. Med. Vet. Entomol..

[13] Viganò R., Aprico J., Besozzi M., Formenti N., Trogu T., Donazzolo C., Obber F., Ferrari N., Lanfranchi P. (2017). Evaluation of pH in game meat of red deer hunted in autumn in the Western Italian Alps. Game Meat Hygiene: Food Safety and Security.

[14] de la Fuente C., San Miguel J.M., Sant M., Alunda J.M., Dominguez I., López A., Carballo M., González A. (2000). Pharyngeal bot flies in *Cervus elaphus* in central Spain: Prevalence and population dynamics. J. Parasitol..

[15] Colwell D.D. (2001). Bot flies and warble flies (order Diptera: Family Oestridae). Parasit. Dis. Wild Mamm..

[16] Ferrazzi P., Rossi L., Demichelis S., Manino A. Morphological, ethological and biochemical characteristics of larvae of Cephenemyia auribarbis (Meigen) and *Pharyngomyia picta* (Meigen). Proceedings of the Proc. XX International Entomology Congress.

[17] Jørgensen D. (2015). Conservation implications of parasite co-reintroduction. Conserv. Biol..

[18] Izdebska J.N., Rolbiecki L., Bielecki W. (2022). Demodex bialoviensis sp. nov. (Acariformes, Demodecidae) a new, specific parasite of the European bison Bison bonasus (Artiodactyla, Bovidae). Int. J. Parasitol. Parasites Wildl..

[19] Perez J.M., Sanchez I., Palma R.L. (2013). The dilemma of conserving parasites: The case of Felicola (Lorisicola) isidoroi (Phthiraptera: Trichodectidae) and its host, the endangered Iberian lynx (Lynx pardinus). Insect Conserv. Divers..

[20] Stringer A.P., Linklater W. (2014). Everything in moderation: Principles of Parasite control for wildlife conservation. Bioscience.

[21] Bezerra-Santos M.A., Moroni B., Mendoza-Roldan J.A., Perrucci S., Cavicchio P., Cordon R., Cianfanelli C., Lia R.P., Rossi L., Otranto D. (2022). Wild carnivores and Thelazia callipaeda zoonotic eyeworms: A focus on wolves. Int. J. Parasitol. Parasites Wildl..

[22] Moroni B., Rossi L., Meneguz P.G., Orusa R., Zoppi S., Robetto S., Marucco F., Tizzani P. (2020). Dirofilaria immitis in wolves recolonizing northern Italy: Are wolves competent hosts?. Parasites Vectors.

[23] Torchin M.E., Lafferty K.D., Dobson A.P., Mckenzie V.J., Kuris A.M. (2003). Introduced species and their missing parasites. Nature.

[24] Bosch F., Manzanell R., Mathis A. (2016). First description of *Onchocerca jakutensis* (Nematoda: Filarioidea) in red deer (*Cervus elaphus*) in Switzerland. Int. J. Parasitol. Parasites Wildl..

[25] Cameron A.E. (1932). The nasal bot fly, Cephenomyia auribarbis Meigen (Diptera, Tachinidae) of the red deer, *Cervus elaphus* L.. Parasitology.

[26] Boch J., Schneidawind H. (1988). Krankheiten des Jagdbaren Wildes: Mit 19 Tabellen.

[27] Gaydou F., Giovo M. Ripopolamento Cervi Anno 2002: Analisi del Primo Semestre (Marzo—Agosto 2002). https://www.catouno.it/wp/wp-content/uploads/2018/06/relazioneIsem.pdf.

[28] Dalmat H.T. (1952). Longevity and further flight range studies on the blackflies (Diptera, Simuliidae), with the use of dye markers. Ann. Entomol. Soc. Am..

[29] Elbers A.R.W., Koenraadt C.J., Meiswinkel R. (2015). Mosquitoes and Culicoides biting midges: Vector range and the influence of climate change. Rev. Sci. Tech..

[30] Király I., Egri B. (2007). Epidemiological characteristics of Cephenemyia stimulator (Clark, 1815) larval infestation in European roe deer (Capreolus capreolus) in Hungary. Acta Zool Acad. Sci. Hung..

[31] Leitner N., Schwarzmann L., Zittra C., Palmieri N., Eigner B., Otranto D., Glawischnig W., Fuehrer H.P. (2016). Morphological and molecular identification of nasopharyngeal bot fly larvae infesting red deer (*Cervus elaphus*) in Austria. Parasitol. Res..

[32] Pedersen A.B., Fenton A. (2015). The role of antiparasite treatment experiments in assessing the impact of parasites on wildlife. Trends Parasitol..

[33] Dougherty E.R., Carlson C.J., Bueno V.M., Burgio K.R., Cizauskas C.A., Clements C.F., Seidel D.P., Harris N.C. (2016). Paradigms for parasite conservation. Conserv. Biol..

